# Single Amino Acid Substitutions Surrounding the Icosahedral Fivefold Symmetry Axis Are Critical for Alternative Receptor Usage of Foot-and-Mouth Disease Virus

**DOI:** 10.3390/v12101147

**Published:** 2020-10-09

**Authors:** Xiao-Hua Gong, Xing-Wen Bai, Ping-Hua Li, Hui-Fang Bao, Meng Zhang, Ying-Li Chen, Pu Sun, Hong Yuan, Lei Huang, Xue-Qing Ma, Yuan-Fang Fu, Yi-Mei Cao, Kun Li, Jing Zhang, Zhi-Yong Li, Dong Li, Zeng-Jun Lu, Zai-Xin Liu

**Affiliations:** State Key Laboratory of Veterinary Etiological Biology, OIE/China National Foot-and-Mouth Disease Reference Laboratory, Lanzhou Veterinary Research Institute, Chinese Academy of Agricultural Sciences, Lanzhou 730046, China; gongxiaohua007@163.com (X.-H.G.); lipinghua@caas.cn (P.-H.L.); baohuifang@caas.cn (H.-F.B.); zhangmeng.ok.2007@163.com (M.Z.); chenyingli@caas.cn (Y.-L.C.); sunpu@caas.cn (P.S.); yzyh1990@126.com (H.Y.); HuangLeiCHAH@163.com (L.H.); maxueqing@caas.cn (X.-Q.M.); fuyuanfang@caas.cn (Y.-F.F.); caoyimei@caas.cn (Y.-M.C.); likun02@caas.cn (K.L.); zhangjing@caas.cn (J.Z.); lizhiyong02@caas.cn (Z.-Y.L.); lidong@caas.cn (D.L.); luzengjun@caas.cn (Z.-J.L.)

**Keywords:** foot-and-mouth disease virus, alternative cellular receptors, site-directed mutations, phenotypic properties, integrin-independent endocytic pathway

## Abstract

The integrins function as the primary receptor molecules for the pathogenic infection of foot-and-mouth disease virus (FMDV) in vivo, while the acquisition of a high affinity for heparan sulfate (HS) of some FMDV variants could be privileged to facilitate viral infection and expanded cell tropism in vitro. Here, we noted that a BHK-adapted Cathay topotype derivative (O/HN/CHA/93tc) but not its genetically engineered virus (rHN), was able to infect HS-positive CHO-K1 cells and mutant pgsD-677 cells. There were one or three residue changes in the capsid proteins of O/HN/CHA/93tc and rHN, as compared with that of their tissue-originated isolate (O/HN/CHA/93wt). The phenotypic properties of a set of site-directed mutants of rHN revealed that E83K of VP1 surrounding the fivefold symmetry axis was necessary for the integrin-independent infection of O/HN/CHA/93tc. L80 in VP2 was essential for the occurrence of E83K in VP1 during the adaptation of O/HN/CHA/93wt to BHK-21 cells. L80M in VP2 and D138G in VP1 of rHN was deleterious, which could be compensated by K83R of VP1 for restoring an efficient infection of integrin-negative CHO cell lines. These might have important implications for understanding the molecular and evolutionary mechanisms of the recognition and binding of FMDV with alternative cellular receptors.

## 1. Introduction

Foot-and-mouth disease virus (FMDV) is the etiological agent of a highly contagious vesicular disease in domestic and wild cloven-hoofed animals, mostly cattle, swine, sheep and goats, and many species of ruminants [1,2]. The virus is the prototypic member that belongs to the genus *Aphthovirus* of the *Picornaviridae* family (http://ictv.global/report/). Seven immunologically and genetically distinguishable serotypes (O, A, C, SAT1–3 and Asia 1) have been reported, and multiple subtypes occur in each serotype with varying geographical distributions [3,4]. The viral genome is a positive-sense, single-stranded RNA approximately 8500 nucleotides in length. The FMDV RNA consists of a single large open reading frame (ORF) flanked by the 5′ and 3′ untranslated regions (UTRs) and a poly(A) tail at its 3′ terminus. There is a small viral protein (termed VPg or 3B) covalently linked to the 5′ terminus of the genomic RNA [5]. The ORF of FMDV encodes a precursor polyprotein that is subsequently cleaved into four structural proteins (VP1–4) and several non-structural proteins (Lpro, 2A, 2B, 2C, 3A, 3B_1–3_, 3Cpro, 3Dpol) [6]. The nonenveloped FMDV virion is assembled from 60 copies each of three surface-exposed capsid proteins VP1–3 and an internal polypeptide VP4, with icosahedral symmetry in a diameter of 27–30 nm [7].

The fundamental initial stage in the life cycle of FMDV is represented by the recognition and binding of receptors on the cell surface that enable virus attachment and entry via the endocytic pathways [8]. αVβ3 has been identified as a common, RGD (arginine-glycine-aspartic acid, 145–147 residues in VP1)-dependent receptor of FMDV [9]. It could be followed by αVβ6, αVβ1, αVβ8 that are also involved in FMDV infection [10,11,12]. The interaction of FMDV with integrin receptors triggers the internalization events via clathrin-mediated endocytosis, the docking of endosomal membrane compartments and trafficking with the acidified endosome vesicles throughout the cells, for the uncoating and release of the viral RNA molecules [13,14,15]. Although αV-integrins serve as the primary receptors for the cytopathic infection of FMDV in vivo [16], the adaptation of FMDV to cultured cells might result in the selection of heparan sulfate (HS)-binding derivatives to establish an efficient infection in vitro [17]. The HS-derived viruses enter cells through the caveola-mediated endocytic pathway [18], whereas some soluble αVβ6-integrin resistants and heparin-sensitive mutants acquire the ability to utilize Jumonji C-domain containing protein 6 (JMJD6) as a third alternative receptor to form clathrin-coated pits [19,20]. In addition, the entry of virus into the cytoplasm of cells can be employed by specific artificial receptors or macropinocytosis for the productive infection of some FMDV serotypes [21,22,23,24,25]. It thus appears that the alteration in receptor utilization of FMDV leads to expanded host range in cell culture, accompanied by critical amino acid substitutions on the outer capsid surface [19,21,26,27].

Remarkably, clusters of conserved mutations at or adjacent to the classical RGD motif in the G–H loop of VP1 (130–165 residues) and compensatory replacements (residues 80 in VP2; 173–175 in VP3; 95–98 in VP1) around the VP1 G–H loop of FMDV would ablate integrin interaction that exhibits the non-RGD binding capacity to infect the target cells (reviewed in [28]). Sa-Carvalho et al. and Borca et al. have representatively described that one or two residue substitutions in VP3 (H56R) and VP2 (E134K) could play a key role in HS binding of FMDV [29,30]. A group of positively charged residue changes (residues 83–85, 108, 110–112 in VP1) surrounding a pore at the icosahedral fivefold axis of the virion might also have great significance for FMDV infection in an RGD- and HS-independent manner (reviewed in [28]).

For our initial study, we were concerned that a genetically engineered virus of Cathay topotype of FMDV serotype O (rHN) with a high affinity for heparin was insufficient to initiate an integrin-independent entry into HS-positive CHO-K1 cells and mutant pgsD-677 cells [31,32]. It was subsequently found that the phenotypic properties of its wild-type (wt) and tissue culture (tc) parental viruses (O/HN/CHA/93wt and O/HN/CHA/93tc) should be distinct from that of rHN in BHK-21 cells and these two integrin-negative CHO cell lines. Thus, the conservative evolution and compensatory effects of several individual residues in the potentially functional regions of the capsid proteins of rHN, O/HN/CHA/93tc and O/HN/CHA/93wt were determined by plaque assays, simulation analysis of virus passages and confocal microscopy in BHK-21, CHO-K1 and pgsD-677 cells. These data argued for the adsorption and penetration of FMDV with alternative cellular receptors, which would contribute to the understanding of FMDV internalization via the integrin-independent endocytic pathway for the infection of different permissive cells in culture.

## 2. Materials and Methods

### 2.1. Cell Lines, Viruses and Plasmids

BSR-T7/5 cells (which can stably express T7 RNA polymerase) were kindly provided by Prof. Karl-Klaus Conzelmann (Max von Pettenkofer-Institute and Gene Center, Munich, Germany) and were cultivated in Glasgow minimal essential medium (GMEM; Gibco) supplemented with 10% fetal bovine serum (FBS; Gibco), 4% tryptose phosphate broth (BD-Bacto) and 1 mg/mL G418 (Sigma). BHK-21 cells were obtained from the China Center for Type Culture Collection (CCTCC; GDC010, Wuhan, China) and were maintained in Dulbecco’s modified Eagle’s medium (DMEM; Gibco) containing 10% FBS and 2 mM L-glutamine (Gibco). Two integrin-negative CHO cell lines (CHO-K1, wild-type, CCL-61; pgsD-677, *N*-acetylglucosaminyl and glucuronyltransferase deficient, CRL-2244) were purchased from the American Type Culture Collection (ATCC; Manassas, VA, USA) and were grown in F-12K nutrient mixture (Gibco) with 10% FBS, 100 U/mL penicillin and 100 μg/mL streptomycin (Sigma). All cells in culture were incubated at 37 °C in a humidified chamber containing 5% CO_2_.

O/HN/CHA/93wt, a tissue-originated isolate of Cathay topotype of FMDV serotype O after six passages in suckling mice, was collected from swine in Zhoukou City of Henan Province of China in 1993 (CNFMDRL, Lanzhou, China). O/HN/CHA/93tc is a cell-adapted derivative after nine passages of BHK-21 cells cytolytically infected with O/HN/CHA/93wt [33]. Additionally, rHN is a genetically engineered virus rescued from an infectious full-length cDNA clone [34], pOFS, which contains the complete genome of O/HN/CHA/93tc with L80M in VP2 and D138G in VP1 deduced from the capsid-coding regions ([35], see Table 1).

### 2.2. Site-Directed Mutagenesis and Transfection

Here, pOFS was used as the original backbone for the construction of site-directed mutated full-length cDNA clones. The introduction of amino acid mutations in the VP2 and VP1 coding regions of FMDV were carried out by one-step overlap extension PCR with five pairs of primers (Table S1), using a QuikChange multisite-directed mutagenesis kit (Stratagene).

BSR-T7/5 cells were seeded in G418-free medium. The plasmid cDNAs were linearized with *Not* I (New England Biolabs), purified by the JetQuick PCR product purification spin kit (Genomed) and transfected into subconfluent monolayers of cultured cells using Lipofectamine 2000 (Invitrogen) according to the manufacturer’s protocol.

### 2.3. Propagation of Virus Progeny and Sequencing of the Capsid-Coding Regions

The supernatants of transfected cells were serially passaged in BHK-21 cells up to 20 times (1/10, V/V), following three freeze–thaw cycles. The total RNAs of the harvested viruses were extracted by using the RNeasy mini kit (Qiagen). The RT-PCR products were amplified with a pair of primers (204: 5′-ACCTCCGACGGGTGGTACGC-3′, NK61: 5′-GACATGTCCTCCTGCATCTG-3′; [36]). The DNA fragments spanning the entire capsid-coding regions of FMDV were purified and confirmed directly by automated sequencing (Liuhe BGI, Beijing, China). The complete nucleotide sequences of O/HN/CHA/93wt were also determined using previously described methods [37].

### 2.4. Plaque Assays

The confluent monolayers of BHK-21, CHO-K1 and pgsD-677 cells were cultured in six-well plates. Serial 10-fold dilutions of virus stocks (200 μL/well) were inoculated onto the surface of cells for 1 h of incubation. Then, the inocula were removed, 2 mL of overlay medium containing 0.6 gum tragacanth (MP Biomedicals) and 1% FBS were added and the cells were routinely incubated at 37 °C in 5% CO_2_. Finally, the cultured cells were fixed with cold acetone/methanol (1:1, V/V) and stained with 0.2% crystal violet (Sigma) at 48 h or 72 h post infection [37,38]. The titer of each virus was evaluated as PFU/mL (plaque forming units, PFU) at least in duplicate per experiment, by counting the number of plaques formed on cell culture monolayers.

### 2.5. Plaque Reduction Assays

The appropriate virus concentrations (10–50 PFU/100 μL) were prepared in DMEM and mixed with equal volumes of soluble heparin sodium (2^x^ mg/mL, x = −4~0; Sigma) or phosphate-buffered saline (PBS, pH = 7.4) at room temperature for 10 min. Monolayers of BHK-21 cells and two CHO cell lines were refreshed with PBS (containing 1 mM CaCl_2_ and 0.5 mM MgCl_2_), pre-treated with an RGD-containing peptide (VR-17: 141-VPNLRGDLQVLAQKVAR-157 [39], Invitrogen; 1 mM), or pre-incubated with JMJD6 polyclonal rabbit antibody (0, 50, 100 μg/mL; Abcam) for 45–60 min at 37 °C. Subsequently, the viral samples were added to the monolayers of cultured cells. The reductions in the average plaque numbers of the selected viruses were measured following the standard plaque assay procedures described above, as compared to those in PBS solutions.

### 2.6. Confocal Microscopy

BHK-21 cells and CHO cell lines on glass-bottom cell culture dishes (20 mm, NEST) were inoculated with the sample preparations of the specific viruses at a multiplicity of infection (MOI) of 10 for 1 h adsorption at 4 °C. The virus suspensions were then removed and the inoculated cells were washed with ice-cold PBS and incubated in fresh medium at 37 °C. At the appropriate times, cells were fixed with 4% paraformaldehyde, permeabilized with 0.1% Tween 20 in PBS and blocked with 1% bovine serum albumin. After washing three times with PBS, the fixed cells were incubated with guinea pig anti-FMDV (serotype O) polyclonal antibody (1:300; CNFMDRL, Lanzhou, China), and clathrin heavy chain monoclonal antibody (1:1000, Thermo Fisher Scientific) or caveolin-1 polyclonal rabbit antibody (1:400, Thermo Fisher Scientific) overnight at 4 °C. The cells were washed again with PBS and incubated with the goat anti-guinea pig IgG H&L (ab150187, Abcam), and goat anti-mouse IgG H&L (ab6785, Abcam) or goat anti-rabbit IgG H&L (ab6939, Abcam) for 1 h at 37 °C. Following immunofluorescence, the antibody-incubated cells were washed with PBS, nuclei-stained with DAPI (1:10,000, Beyotime) for 5 min at room temperature, then washed, mounted and viewed under a Leica TCS SP8 confocal microscope. The images were captured digitally and processed by using Adobe Photoshop software.

## 3. Results

### 3.1. E83K in VP1 is Responsible for the Alteration in Cellular Receptor Recognition of O/HN/CHA/93tc to Establish an Efficient Infection in Integrin-Negative CHO Cell Lines

**Figure 1:**
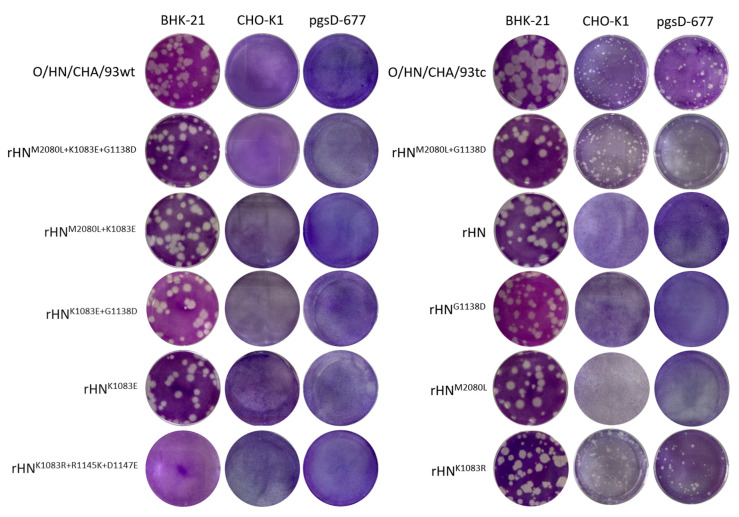
The plaque phenotypes of site-directed mutants of rHN on BHK-21 cells and two CHO cell lines. The procedures of plaque assays for each virus were done as described in the Materials and Methods. O/HN/CHA/93wt and O/HN/CHA/93tc could be regarded in parallel as controls.

The mice-originated isolate (O/HN/CHA/93wt) and its BHK-adapted derivative (O/HN/CHA/93tc) of Cathay topotype of FMDV serotype O were used to perform plaque assays on BHK-21, CHO-K1 and pgsD-677 cells. As a result, O/HN/CHA/93wt was unable to produce plaques on HS-positive CHO-K1 cells and mutant pgsD-677 cells, whereas O/HN/CHA/93tc formed plaques on these two integrin-negative CHO cell lines (Figure 1). Comparative analysis of the complete genomic sequences of O/HN/CHA/93wt and O/HN/CHA/93tc showed only one amino acid substitution (E83K in VP1) in the capsid-coding regions (Table 1). In this case, site-directed (reverse) mutations were introduced into the VP2 and VP1 coding regions of pOFS, for the construction of two infectious full-length genome-modified cDNA clones that contain the whole capsid-coding regions of O/HN/CHA/93wt (M80L in VP2, K83E and G138D in VP1) and O/HN/CHA/93tc (M80L in VP2, G138D in VP1), in terms of being 100% identical at the amino acid level (Table 2). The infectious phenotypes of transfected supernatants from BSR/T7-5 cells (rHN^M2080L+K1083E+G1138D^ and rHN^M2080L+G1138D^) were examined by standard plaque assays on BHK-21 cells and two CHO cell lines. As expected, the formation of plaques was observed on CHO-K1 and pgsD-677 cells infected with rHN^M2080L+G1138D^, but not rHN^M2080L+K1083E+G1138D^ (Figure 1). In addition, the results from plaque reduction assays showed that O/HN/CHA/93wt and rHN^M2080L+K1083E+G1138D^, but not O/HN/CHA/93tc and rHN^M2080L+G1138D^, could be almost completely abolished by the RGD-containing peptide VR-17 for efficient infection of BHK-21 cells (Table 2). Therefore, it seems that the presence of E83K in VP1 would lead to the initiation of infection of O/HN/CHA/93tc and rHN^M2080L+G1138D^ in cultured cells in an integrin-independent manner.

### 3.2. L80 in VP2 is Involved in the Occurrence of E83K in VP1 during the Adaptation of O/HN/CHA/93wt to BHK-21 Cells

Subsequently, the transfected viral supernatants were then serially passaged in BHK-21 cells, and E83K of VP1 was detected in cell-passaged derivatives of rHN^M2080L+K1083E+G1138D^ (10th, Table 3). Consequently, to simulate the adaptive evolution process of O/HN/CHA/93wt in BHK-21 cells, the other three site-directed mutants were generated (rHN^K1083E+G1138D^, rHN^M2080L+K1083E^ and rHN^K1083E^; Table 2). It is intriguing that E83K appeared only in VP1 of rHN^M2080L+K1083E^ after eight passages of BHK-21 cells (Table 3). These results suggested that the co-evolution of L80 in VP2 and E83K in VP1 might have important implications for the recognition and binding of O/HN/CHA/93tc with alternative receptors on the surface of cells in culture.

### 3.3. Both L80M in VP2 and D138G in VP1 Are Detrimental for the Infection of rHN in Two CHO Cell Lines

As already previously described, E83K was displayed in VP1 of rHN, however, no plaques were produced by this backbone virus on CHO-K1 and pgsD-677 cells [31]. There were only two amino acid differences in the capsid-coding regions (L80M in VP2, D138G in VP1) between O/HN/CHA/93tc (rHN^M2080L+G1138D^) and rHN (Table 1, see Materials and Methods). To identify the molecular determinant(s) for the non-infectious phenotypes of rHN in these two CHO cell lines, rHN^M2080L^ and rHN^G1138D^ were generated from BSR/T7-5 cells by the transfection of the expectant site-directed mutated plasmid constructions (Table 2). None of them could acquire the ability to form plaques on CHO-K1 and pgsD-677 cells (Figure 1). Nonetheless, these two site-directed mutants retained the non-RGD binding capacity for efficient infection in BHK-21 cells (Table 2). These results demonstrated that L80 in VP2 and D138 in VP1 of O/HN/CHA/93tc were essential to facilitate the non-integrin-dependent pathway in two CHO cell lines.

### 3.4. K83R in VP1 of rHN Plays a Functional Role to Expand Virus Tropism to Cell Types

**Figure 2:**
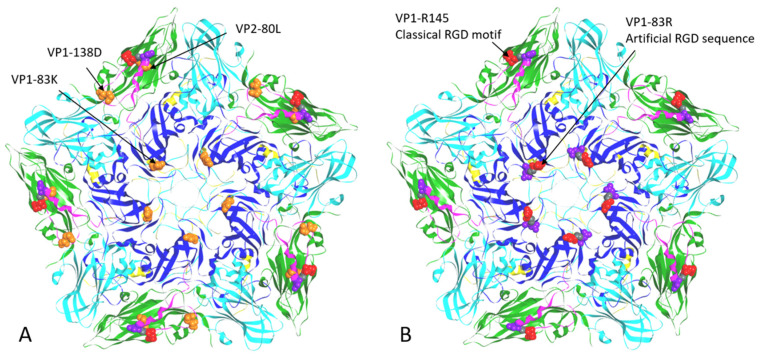
Locations of site-directed mutations in one of the twelve pentamers of (**A**) rHN^M2080L+G1138D^ and (**B**) rHN^K1083R^. The crystallographic coordinates of O1BFS (1FOD) were used for molecular modeling [40]. The ribbon diagram of a pentamer composed of five copies of protomers is shown with the respective color codes of VP1 (blue), VP2 (green), VP3 (cyan) and VP4 (yellow, internal). The conformation of the VP1 G–H loop (130–165 residues) is highlighted in magenta. The specific amino acid residues at positions 80 (Leu, orange) in VP2, as well as 83 (Lys, orange) and 138 (Asp, orange) in VP1 of rHN^M2080L+G1138D^, the artificial RGD sequence (83–85 residues, red-grey-purple) and the classical RGD motif (145–147 residues, red-grey-purple) in VP1 of rHN^K1083R^, are labeled as space-filling models.

As also mentioned in our previous and present studies, although rHN, rHN^M2080L^ and rHN^G1138D^ were unable to induce plaques on two CHO cell lines, the pre-treatment with the VR-17 RGD-containing peptide had no influence on the formation of viral plaques on BHK-21 cells ([31,32], Table 2). Consequently, two site-directed mutated full-length cDNA clones were constructed by the introduction of K83R (KGD→RGD, 83–85 residues) as well as K83R, R145K and D147E (RGD→KGE) in the VP1 coding region of pOFS, respectively (Table 2). Unfortunately, no infectious progeny virus was detected from the corresponding supernatants of rHN^K1083R+R1145K+D1147E^ after transfection of its *Not* I-linearized plasmid cDNAs into BSR/T7-5 cells (Figure 1). In the VR-17 inhibition assay, rHN^K1083R^ maintained the non-RGD binding capacity to infect BHK-21 cells (Table 2). Interestingly, the results from plaque assays manifested that K83R of VP1 (rHN^K1083R^) could compensate the deleterious effects of L80M in VP2 and D138G in VP1 of rHN for the production of viral plaques on CHO-K1 and pgsD-677 cells (Figure 1). It was speculated that rHN^K1083R^ was unable to resume the involvement of the integrin-dependent signaling pathway in BHK-21 cells but could acquire the plaque-forming ability on two CHO cell lines. Moreover, site-directed mutations in the classical RGD motif were particularly deleterious for the virus (rHN^K1083R+R1145K+D1147E^, non-infectious; Table 2, Figure 2), despite an artificial RGD sequence located upstream of the VP1 G-H loop.

### 3.5. The Site-Directed Mutants of rHN with a High Affinity for Heparin Allow Caveolin-Mediated Endocytosis in Cultured Cells

**Figure 3:**
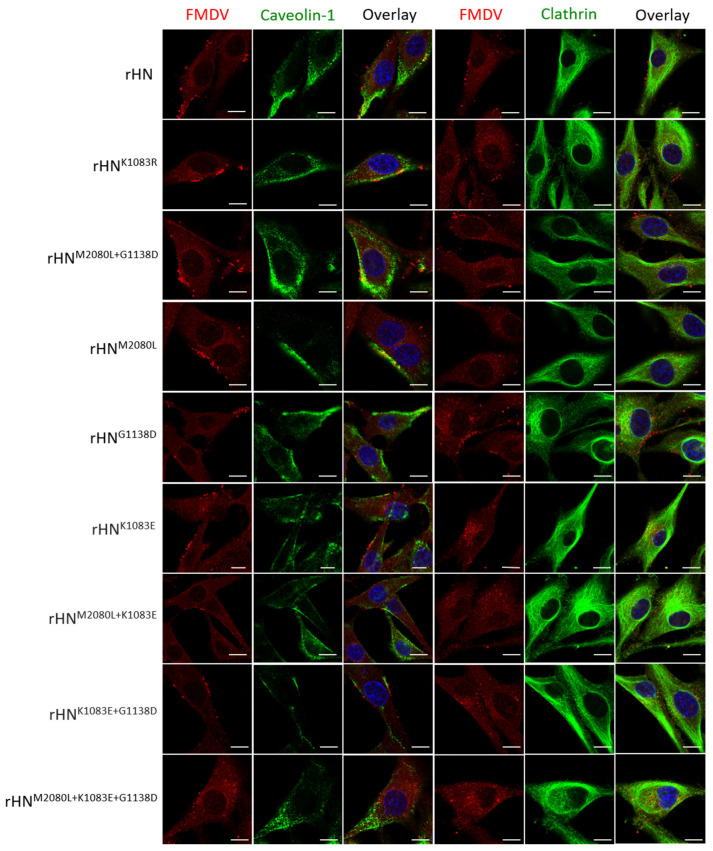
Interactions of site-directed mutants of rHN with clathrin and caveolin-1 in BHK-21 cells. The infection of BHK-21 cells with each virus (10 MOI) for the study of the internalization events was examined by immunofluorescence and confocal microscopy, as described in the Materials and Methods. At 15 min after the incubation temperature was raised from 4 °C to 37 °C, the cells were fixed and stained for virus (red) and either clathrin (green) or caveolin-1 (green) with the appropriate antibodies. Scale bars = 10 μm.

**Figure 4:**
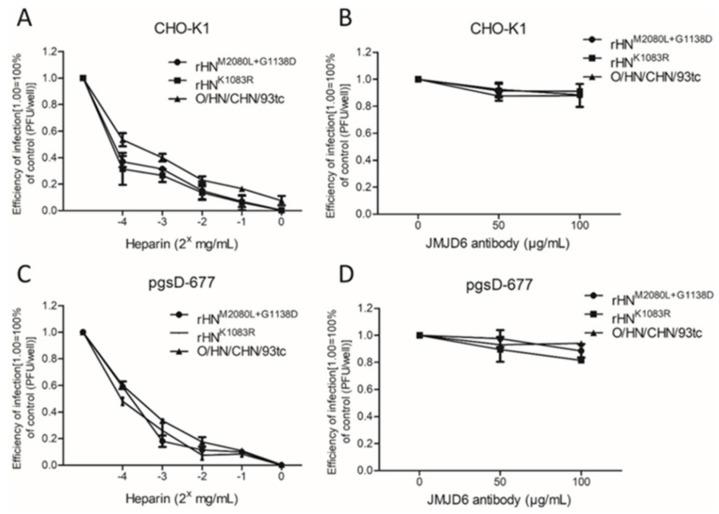
Effects of heparin and JMJD6 antibody on the infectivity of the specific FMDVs in two integrin-negative CHO cell lines. The plaque reduction assays were performed after the neutralization of FMDV with heparin (2^x^ mg/mL, x = −4~0) or by pre-treatment with blocking antibodies to JMJD6 (0, 50, 100 μg/mL) on (**A**,**B**) CHO-K1 and (**C**,**D**) pgsD-677 cells.

**Figure 5:**
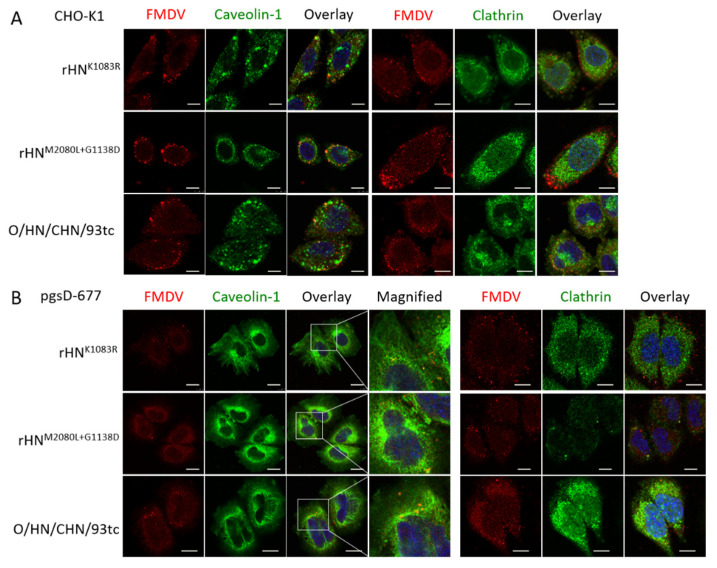
Analysis of the endocytic pathway of the specific FMDVs in (**A**) CHO-K1 and (**B**) pgsD-677 cells. The detailed procedures of immunofluorescence and confocal microscopy for the entry of O/HN/CHA/93tc, rHN^M2080L+G1138D^ and rHN^K1083R^ into these two non-integrin CHO cell lines were processed as described in the Materials and Methods. Virus (red), and clathrin (green) or caveolin-1 (green) upon entering the cell were probed after a shift to 37 °C for 15 min. Scale bars = 10 μm.

Of the eight site-directed mutants of rHN, the plaque numbers of rHN^K1083E^, rHN^M2080L+1083E^, rHN^K1083E+G1138D^ and rHN^M2080L+K1083E+G1138D^ formed on BHK-21 cells were effectively reduced by the VR-17 RGD-containing peptide rather than heparin (Table 2). The entry of these four RGD-integrin binding viruses into BHK-21 cells was dependent on the clathrin-mediated endocytic pathway, whereas the other five rescued viruses (including rHN) with a high affinity for heparin could enter BHK-21 cells in a caveolin-mediated manner (Table 2, Figure 3). It has already been shown that rHN^K1083R^ and rHN^M2080L+G1138D^, two of these five heparin-sensitive viruses, acquired the plaque-forming ability on two integrin-negative CHO cell lines (see Figure 1). The number of plaques formed by rHN^K1083R^ and rHN^M2080L+G1138D^ on CHO-K1 and pgsD-677 cells was significantly reduced by the addition of heparin, and yet the pre-incubation with JMJD6 antibodies had little impact on the number of plaques produced by these two site-directed mutants (Figure 4). As a matter of fact, rHN^K1083R^ and rHN^M2080L+G1138D^ were extensively colocalized with caveolin, but there was slight colocalization of clathrin in not only HS-positive CHO-K1 cells but also mutant pgsD-677 cells (Figure 5). These results offered an insight into the internalization of a cell-adapted Cathay topotype virus and its genetically engineered FMDV variants with a high affinity for heparin and non-RGD binding capacity, for the infection of cultured cells.

## 4. Discussion

The flexibility of non-RGD-dependent receptor usage reflects quasispecies dynamics of FMDV populations within various tissue-specific cells from susceptible host species [41,42,43,44]. The efficiency of the primary and alternative receptor utilization of FMDV might be related to the viral serotype, due to the structural differences on the capsid surface [45,46,47]. It has been characterized that the representatives of FMDV serotype O (O_1_Camp and O/TAW/2/99) bind to soluble αVβ6 with relatively higher affinity than αVβ3 for virus adsorption and penetration [48]. The ligand-binding domain of the subunit of β6 appears to contribute to the internalization of the virus [47,49], but there was no detectable level of the β6 subunit by western blotting analysis in BHK-21 cell lysates [38,50]. Therefore, it would be understandable for the alteration in the receptor recognition and binding of a cell-adapted Cathay topotype derivative (O/HN/CHA/93tc) of FMDV serotype O to establish an efficient infection in BHK-21, CHO-K1 and pgsD-677 cells (Figure 1, Table 2).

The distinct phenotypic properties of rHN, rHN^M2080L^ and rHN^G1138D^ in the integrin-independent infection of BHK-21 and CHO cell lines (Figure 1, Table 2) might be influenced by the differences of disaccharide sequences and binding properties of the cell-specific HS species [51]. This in turn could be helpful to explain the 10- to 50-fold decrease in the titers of rHN^K1083R^ and rHN^M2080L+G1138D^ in pgsD-677 cells (comparable to that in CHO-K1 cells, Table 2). Despite this, the results from plaque reduction assays and confocal microscopy forced us to reconsider the capacity of these two heparin-sensitive viruses for the efficient infection of cultured cells in an integrin-independent manner (Table 2, Figure 3, Figure 4, Figure 5). The cumulative findings in non-RGD-containing variants of FMDV serotypes O, A, C have proposed that the positively charged residues at receptor-related protein-binding sites might also act as one of the molecular determinants for JMJD6-mediated infection of pgsD-677 cells [52,53]. The possibility of lipid raft-dependent macropinocytosis was eliminated by the attachment of heparin-sensitive mutants (Figure S1) and the activation of dextran uptake by non-integrin-binding variants (Figure S2) on the surface of JMJD6-positive CHO cells. These seemingly puzzling results could no doubt further enrich our knowledge of early internalization events in the non-integrin-dependent cell entry of FMDV.

The nucleotide differences in the complete genomic sequences of O/HN/CHA/93wt, O/HN/CHA/93tc and rHN provided evidence of the evolutionary fitness in a broad spectrum of extinction escape viral mutants (Table 1). It has been widely accepted that E1083K in VP1 might be one of the dominant determinants for the non-RGD binding capacity of FMDV during cell adaptation [31,54,55,56]. The results from successive passages of site-directed mutants in BHK-21 cells retrospectively reminded us that the VP2 and VP1 coding sequences of rHN were not thought to be amplified from the same cDNA templates (Table 3, [35]). Molecular modeling has revealed that two functionally defined capsid residues at positions 80 of VP2 (L80M) and 138 of VP1 (D138G) were located far away from the primary mutation (E83K in VP1), but somewhere nearby the classical RGD motif in the G–H loop of VP1 (Figure 2, [31,32]). By this token, it was to be regretted but came as no surprise that none of the infectious RGD- and HS-independent viruses could be rescued successfully by the replacements of R145K and D147E in VP1 of rHN for the infection of two CHO cell lines (Table 2).

Indeed, the most comparable other example was O/CHA/90, an inactivated vaccine candidate of Cathay topotype of FMDV serotype O [57]. E83K and the other three interfacial residues (H108Y, R172, T174F/Y) in VP1 surrounding the fivefold axis of the virion appear to be implicated in the altered receptor recognition of its BHK-adapted derivative (vac-O/CHA/90) for the infection of cultured cells and animals [54]. H108 and T174 were mapped in VP1 of O/HN/CHA/93tc and, instead, the conserved cysteine residues co-existing elsewhere on the FMDV capsid (C2078 and C2130) could tolerate change in the flexibility of the VP1 G–H loop for modulating antigenicity and cell adaptation capacity (Table S2). Clusters of ligand residues overlapping antigenic sites still participate in the compatibility of FMDV with moderate infectivity in tissue culture, by stereo- and electro-chemical modification in ionic, polar and sulfur groups (reviewed in [28]). As compared with that of O/HN/CHA/93tc, rHN had a relatively lower virulence with a partial loss of antigenicity in vivo [34,35]. Accordingly, the potential implications of conservative and compensatory evolution in the functional capsid regions of rHN should be and have already been taken into account for developing anti-FMDV vaccine candidates with improved immunogenicity and stability [34,58,59], and companion diagnostic preparations [60].

## 5. Conclusions

In general, two positively charged residues (E83K/K83R in VP1) surrounding the pentamer axis were crucial in the response to the integrin-independent infection of a few genetic and engineered FMDVs (Cathay topotype, serotype O) in BHK-21 and CHO cell lines. The presence of E83K in VP1 might have co-evolved with L80 in VP2, and the introduction of K83R in VP1 might compensate for the deleterious effects of L80M in VP2 and D138G in VP1 upon the interaction of FMDV with alternative cellular receptors. These data prompt efforts to interpret the alternative receptor usage of FMDV involved in genetic heterogeneity, viral pathogenesis and antigenic variation.

## Figures and Tables

**Table 1:** Comparative analysis of the complete genomic sequences of O/HN/CHA/93wt, O/HN/CHA/93tc and its genetically engineered virus (rHN) ^a^.

Genomic Region	Position ^b^	Virus		
		O/HN/CHA/93wt	O/HN/CHA/93tc ^c^	rHN ^d^
S	(353)	(T)	(C)	(C)
	(361)	(A)	(G)	(G)
IRES	(1–5)	(A_5_)	(A_5_)	(A_6_) ^£^
	(279)	(C)	(A/C) *	(C)
Lpro	24	Arg (CGA)	Arg (CGA)/**Gln** (CAA) ^#^	Arg (CGA)
	87	Glu (GAA)	Glu (GAA)/**Lys** (AAA) ^#^	Glu (GAA)
	172	Pro (CCA)	Pro (CCA)	Pro (CCG)
	173	Asp (GAC)	Asp (GAC)	Asp (GAT)
VP2	80	Leu (CTG)	Leu (CTG)	**Met** (ATG)
VP3	57	Phe (TTC)	Phe (TTC/TTT) ^$^	Phe (TTC)
	138	Ala (GCG)	Ala (GCG)	Ala (GCC)
	153	Asn (AAT)	Asn (AAT)	Asn (AAC)
	201	Val (GTG)	Val (GTG)	Val (GTT)
VP1	83	Glu (GAG)	**Lys** (AAG)	**Lys** (AAG)
	138	Asp (GAC)	Asp (GAC)	**Gly** (GGC)
2B	107	Ile (ATC)	Ile (ATC)	Ile (ATA)

**^a^** The nucleotide differences in the small (S) fragment and the element of internal ribosome entry site (IRES) of the 5′-UTR, and triple-code codons for each one deduced in the leader proteinase (Lpro), VP1–3 and 2B coding regions are given in parentheses. The nucleotide mutations and amino acid substitutions (three-letter abbreviations) in the genomic RNA molecules of O/HN/CHA/93tc and rHN are indicated in underline and bold formats, respectively. **^b^** The positions of nucleotides for each of the viral genes (in parentheses) and amino acid residues for each protein are independently numbered, referring to O/HN/CHA/93wt. **^c^** Mixtures of (*) nucleotides, (^#^) non-synonymous and (^$^) synonymous codons are displayed in the O/HN/CHA/93tc genome. **^d^** An adenine nucleotide (^£^) was accidentally inserted at the 5′ terminus of IRES, while those of six silent mutations were intentionally introduced for the construction of an infectious cDNA of rHN [35].

**Table 2:** The phenotypic properties of distinct genetic and engineered foot-and-mouth disease viruses (FMDVs) in BHK-21 and two CHO cell lines.

Virus ^a^	Individual Amino Acid Residues ^b^				Virus Titer (PFU/mL) ^c^			Inhibition of Viral Infection in BHK-21 Cells ^d^	
	VP2	VP1			BHK-21	CHO-K1	pgsD-677	Heparin	VR-17
	80	83–85	138	145–147					
* O/HN/CHN/93wt	L	EGD	D	RGD	2.5 × 10^7^	<5	<5	0.5	98
^#^ O/HN/CHN/93tc	L	KGD	D	RGD	7.0 × 10^7^	2.4 × 10^4^	5.5 × 10^2^	97	0.2
rHN	M	KGD	G	RGD	1.1 × 10^7^	<5	<5	85	0.2
rHN^M2080L^	L	KGD	G	RGD	3.7 × 10^7^	<5	<5	80	0.5
rHN^G1138D^	M	KGD	D	RGD	2.5 × 10^7^	<5	<5	76	0.7
^#^ rHN^M2080L+G1138D^	L	KGD	D	RGD	6.8 × 10^7^	3.2 × 10^4^	6.0 × 10^2^	98	0.2
rHN^K1083E^	M	EGD	G	RGD	4.2 × 10^7^	<5	<5	0.2	98
rHN^M2080L+K1083E^	L	EGD	G	RGD	1.8 × 10^7^	<5	<5	0.4	86
rHN^K1083E+G1138D^	M	EGD	D	RGD	3.0 × 10^7^	<5	<5	0.5	96
* rHN^M2080L+K1083E+G1138D^	L	EGD	D	RGD	5.2 × 10^7^	<5	<5	0.3	96
rHN^K1083R^	M	RGD	G	RGD	5.0 × 10^7^	1.5 × 10^3^	1.2 × 10^2^	95	0.4
^$^ rHN^K1083R+R1145K+D1147E^	M	RGD	G	KGE	<5	<5	<5	—	—

**^a^** A detailed description for the generation of O/HN/CHA/93wt, O/HN/CHA/93tc, rHN and its site-directed mutants was provided in the Materials and Methods. The site-directed mutants of rHN are designated with the superscripts of the original (left) and introduced (right) amino acid residues in the capsid-coding regions. The distribution of amino acid substitutions in the FMDV capsid is denoted by a four-digit numbering system. The first and last three digits represent the capsid proteins (2, VP2; 1, VP1) and corresponding positions occupied by specific amino acid residues in VP2 and VP1, respectively. The predicted amino acid sequences in the capsid-coding regions of (*) O/HN/CHA/93wt and rHN^M2080L+K1083E+G1138D^ as well as (^#^) O/HN/CHA/93tc and rHN^M2080L+G1138D^ are 100% identical to each other. No infectious progeny virus of (^$^) rHN^K1083R+R1145K+D1147E^ was detectable by the secondary introduction of R145K and D147E in the classical RGD motif of rHN^K1083R^. ^b^ One-letter amino acid codes are used. **^c^** The titer of each virus was determined by plaque assays on BHK-21, CHO-K1 and pgsD-677 cells (<5, no plaques). **^d^** The inhibition ratio of FMDV infection (%) by heparin (0.5 mg/mL) and VR-17 (1 mM) was analyzed by plaque reduction assays in BHK-21 cells (comparable to that in PBS solutions; —, not done).

**Table 3:** The sequence divergence in the entire capsid-coding regions of site-directed mutants of rHN after serial passages in BHK-21 cells **^a^**.

Virus	Original Mutation			Acquired Mutation ^b^	No. of Passages
	VP2	VP1			
rHN	—	—	—	NC	20
rHN^M2080L^	M80L	—	—	NC	20
rHN^K1083E^	—	K83E	—	NC	20
rHN^K1083R^	—	K83R	—	NC	11
rHN^G1138D^	—	—	G138D	NC	20
rHN^M2080L+K1083E^	M80L	K83E	—	E83K in VP1	8
rHN^M2080L+G1138D^	M80L	—	G138D	NC	20
rHN^K1083E+G1138D^	—	K83E	G138D	NC	20
rHN^M2080L+K1083E+G1138D^	M80L	K83E	G138D	E83K in VP1	10

**^a^** The transfected viral supernatants were successively passaged at a multiplicity of infection (MOI) of ≈1.0 (1/10, V/V) up to 20 times in BHK-21 cells. **^b^** A single amino acid substitution (E83K in VP1) was found in the capsid-coding regions of rHN^M2080L+K1083E^ and rHN^M2080L+K1083E+G1138D^. NC, no change in amino acid sequences of the viral capsid proteins.

## Supplementary Materials

The following are available online at https://www.mdpi.com/1999-4915/12/10/1147/s1, Figure S1: The attachment of FMDV on the surface of CHO cell lines, Figure S2: The uptake of dextran stimulated by FMDV internalization into CHO cell lines, Table S1: The primer pairs used for the construction of the full-length genome-modified cDNA clones of FMDV, Table S2: Comparison of the deduced amino acid sequences in the capsid coding regions of O/CHA/90, O/HN/CHA/93wt, and their derivatives.

## References

[1] Knight-Jones T.J.D., Robinson L., Charleston B., Rodriguez L.L., Gay C., Sumption K.J., Vosloo W. (2016). Global foot-and-mouth disease research update and gap analysis: Epidemiology, wildlife and economics. Transbound. Emerg. Dis..

[2] Shanafelt D., Perrings C. (2017). Foot and mouth disease: The risks of the international trade in live animals. Rev. Sci. Tech. l’OIE.

[3] Freimanis G., Di Nardo A., Bankowska K., King D., Wadsworth J., Knowles N., King D. (2016). Genomics and outbreaks: Foot and mouth disease. Rev. Sci. Tech. l’OIE.

[4] Brito B.P., Rodriguez L.L., Hammond J.M., Pinto J., Perez A.M. (2015). Review of the global distribution of foot-and-mouth disease virus from 2007 to 2014. Transbound. Emerg. Dis..

[5] Martinez-Salas E., Belsham G.J., Sobrino F., Domingo E. (2017). Genome organization, translation and replication of foot-and-mouth disease virus RNA. Foot-and-Mouth Disease Virus: Current Research and Emerging Trends.

[6] Herod M.R., Gold S., Lasecka-Dykes L., Wright C., Ward J.C., McLean T.C., Forrest S., Jackson T., Tuthill T.J., Rowlands D.J. (2017). Genetic economy in picornaviruses: Foot-and-mouth disease virus replication exploits alternative precursor cleavage pathways. PLoS Pathog..

[7] Jiang P., Liu Y., Ma H.-C., Paul A.V., Wimmer E. (2014). Picornavirus morphogenesis. Microbiol. Mol. Biol. Rev..

[8] Ruiz-Saenz J., Goez Y., Tabares W., López-Herrera A. (2009). Cellular receptors for foot and mouth disease virus. Intervirology.

[9] Berinstein A., Roivainen M., Hovi T., Mason P.W., Baxt B. (1995). Antibodies to the vitronectin receptor (integrin alpha V beta 3) inhibit binding and infection of foot-and-mouth disease virus to cultured cells. J. Virol..

[10] Jackson T., Sheppard D., Denyer M., Blakemore W., King A.M. (2000). The epithelial integrin alphavbeta6 is a receptor for foot-and-mouth disease virus. J. Virol..

[11] Jackson T., Mould A.P., Sheppard D., King A.M. (2002). Integrin alphavbeta1 is a receptor for foot-and-mouth disease virus. J. Virol..

[12] Jackson T., Clark S., Berryman S., Burman A., Cambier S., Mu D., Nishimura S., King A.M. (2004). Integrin alphavbeta8 functions as a receptor for foot-and-mouth disease virus: Role of the beta-chain cytodomain in integrin-mediated infection. J. Virol..

[13] O’Donnell V., Larocco M., Duque H., Baxt B. (2005). Analysis of foot-and-mouth disease virus internalization events in cultured cells. J. Virol..

[14] Berryman S., Clark S., Monaghan P., Jackson T. (2005). Early events in integrin alphavbeta6-mediated cell entry of foot-and-mouth disease virus. J. Virol..

[15] Martín-Acebes M.A., González-Magaldi M., Sandvig K., Sobrino F., Armas-Portela R. (2007). Productive entry of type C foot-and-mouth disease virus into susceptible cultured cells requires clathrin and is dependent on the presence of plasma membrane cholesterol. Virology.

[16] Neff S., Sá-Carvalho D., Rieder E., Mason P.W., Blystone S.D., Brown E.J., Baxt B. (1998). Foot-and-mouth disease virus virulent for cattle utilizes the integrin αvβ3 as its receptor. J. Virol..

[17] Jackson T., Ellard F.M., Ghazaleh R.A., Brookes S.M., Blakemore W.E., Corteyn A.H., Stuart D.I., Newman J.W., King A.M. (1996). Efficient infection of cells in culture by type O foot-and-mouth disease virus requires binding to cell surface heparan sulfate. J. Virol..

[18] O׳donnell V., Larocco M., Baxt B. (2008). Heparan sulfate-binding foot-and-mouth disease virus enters cells via caveola-mediated endocytosis. J. Virol..

[19] Lawrence P., Rai D., Conderino J.S., Uddowla S., Rieder E. (2016). Role of Jumonji C-domain containing protein 6 (JMJD6) in infectivity of foot-and-mouth disease virus. Virology.

[20] Lawrence P., Pacheco J.M., Stenfeldt C., Arzt J., Rai D.K., Rieder E. (2016). Pathogenesis and micro-anatomic characterization of a cell-adapted mutant foot-and-mouth disease virus in cattle: Impact of the Jumonji C-domain containing protein 6 (JMJD6) and route of inoculation. Virology.

[21] Mason P.W., Rieder E., Baxt B. (1994). RGD sequence of foot-and-mouth disease virus is essential for infecting cells via the natural receptor but can be bypassed by an antibody-dependent enhancement pathway. Proc. Natl. Acad. Sci. USA.

[22] Baxt B., Mason P.W. (1995). Foot-and-mouth disease virus undergoes restricted replication in macrophage cell cultures following fc receptor-mediated adsorption. Virology.

[23] Mason P., Berinstein A., Baxt B., Parsells R., Kang A., Rieder E. (1996). Cloning and expression of a single-chain antibody fragment specific for foot-and-mouth disease virus. Virology.

[24] Rieder E., Berinstein A., Baxt B., Kang A., Mason P.W. (1996). Propagation of an attenuated virus by design: Engineering a novel receptor for a noninfectious foot-and-mouth disease virus. Proc. Natl. Acad. Sci. USA.

[25] Han S.-C., Guo H.-C., Sun S.-Q., Jin Y., Wei Y.-Q., Feng X., Yao X.-P., Cao S.-Z., Liu D.X., Liu X.-T. (2016). Productive entry of foot-and-mouth disease virus via macropinocytosis independent of phosphatidylinositol 3-kinase. Sci. Rep..

[26] Fry E.E., Lea S.M., Jackson T., Newman J.W., Ellard F.M., Blakemore W.E., Abu-Ghazaleh R., Samuel A., King A.M., Stuart D.I. (1999). The structure and function of a foot-and-mouth disease virus-oligosaccharide receptor complex. EMBO J..

[27] Fry E.E., Newman J.W.I., Curry S., Najjam S., Jackson T., Blakemore W., Lea S.M., Miller L., Burman A., King A.M.Q. (2005). Structure of foot-and-mouth disease virus serotype A1061 alone and complexed with oligosaccharide receptor: Receptor conservation in the face of antigenic variation. J. Gen. Virol..

[28] Dill V., Eschbaumer M. (2019). Cell culture propagation of foot-and-mouth disease virus: Adaptive amino acid substitutions in structural proteins and their functional implications. Virus Genes.

[29] Sa-Carvalho D., Rieder E., Baxt B., Rodarte R., Tanuri A., Mason P.W. (1997). Tissue culture adaptation of foot-and-mouth disease virus selects viruses that bind to heparin and are attenuated in cattle. J. Virol..

[30] Borca M., Pacheco J.M., Holinka L.G., Carrillo C., Hartwig E.J., Garriga D., Kramer E., Rodriguez L., Piccone M.E. (2012). Role of arginine-56 within the structural protein VP3 of foot-and-mouth disease virus (FMDV) O1 Campos in virus virulence. Virology.

[31] Bai X., Bao H., Li P., Wei W., Zhang M., Sun P., Cao Y., Lu Z., Fu Y., Xie B. (2014). Effects of two amino acid substitutions in the capsid proteins on the interaction of two cell-adapted PanAsia-1 strains of foot-and-mouth disease virus serotype O with heparan sulfate receptor. Virol. J..

[32] Bai X.-W., Bao H.-F., Li P.-H., Ma X.-Q., Sun P., Bai Q.-F., Zhang M., Yuan H., Chen D.-D., Li K. (2019). Engineering responses to amino acid substitutions in the VP0-and VP3-coding regions of PanAsia-1 strains of foot-and-mouth disease virus serotype O. J. Virol..

[33] Cao Y.-M., Lu Z.-J., Sun P., Fu Y., Tian F., Hao X., Bao H.-F., Liu X.-T., Liu Z. (2011). A pseudotype baculovirus expressing the capsid protein of foot-and-mouth disease virus and a T-Cell immunogen shows enhanced immunogenicity in mice. Virol. J..

[34] Li P.-H., Bai X.-W., Sun P., Li D., Lu Z.-J., Cao Y.-M., Fu Y.-F., Bao H.-F., Chen Y.-L., Xie B.-X. (2012). Evaluation of a genetically modified foot-and-mouth disease virus vaccine candidate generated by reverse genetics. BMC Veter. Res..

[35] Cao W., Li P., Bai X., Lu Z., Sun P., Liu Z. (2010). Rescue and identification of virus activity of foot-and-mouth disease virus strain O/HN/93 from full length cDNA clone. Acta Agric. Boreali-Sinica.

[36] Knowles N., Samuel A.R., Davies P.R., Midgley R.J., Valarcher J.-F. (2005). Pandemic strain of foot-and-mouth disease virus serotype O. Emerg. Infect. Dis..

[37] Bai X., Bao H., Li P., Sun P., Kuang W., Cao Y., Lu Z., Liu Z., Liu X. (2010). Genetic characterization of the cell-adapted PanAsia strain of foot-and-mouth disease virus O/Fujian/CHA/5/99 isolated from swine. Virol. J..

[38] Lawrence P., Larocco M., Baxt B., Rieder E. (2013). Examination of soluble integrin resistant mutants of foot-and-mouth disease virus. Virol. J..

[39] Núñez J.I., Molina N., Baranowski E., Domingo E., Clark S., Burman A., Berryman S., Jackson T., Sobrino F. (2007). Guinea pig-adapted foot-and-mouth disease virus with altered receptor recognition can productively infect a natural host. J. Virol..

[40] Logan D., Abu-Ghazaleh R., Blakemore W., Curry S., Jackson T., King A., Lea S., Lewis R., Newman J., Parry N. (1993). Structure of a major immunogenic site on foot-and-mouth disease virus. Nat. Cell Biol..

[41] Ruiz-Jarabo C.M., Sevilla N.N., Dávila M., Gómez-Mariano G., Baranowski E., Domingo E. (1999). Antigenic properties and population stability of a foot-and-mouth disease virus with an altered Arg-Gly-Asp receptor-recognition motif. J. Gen. Virol..

[42] Baranowski E., Ruiz-Jarabo C.M., Sevilla N., Andreu D., Beck E., Domingo E. (2000). Cell recognition by foot-and-mouth disease virus that lacks the RGD integrin-binding motif: Flexibility in aphthovirus receptor usage. J. Virol..

[43] Baranowski E., Ruiz-Jarabo C.M., Domingo E. (2001). Evolution of cell recognition by viruses. Science.

[44] Li P.-H., Lu Z.-J., Bao H.-F., Li D., King D.P., Sun P., Bai X.-W., Cao W., Gubbins S., Chen Y.-L. (2011). In-vitro and in-vivo phenotype of type Asia 1 foot-and-mouth disease viruses utilizing two non-RGD receptor recognition sites. BMC Microbiol..

[45] Mateu M.G. (2017). The foot-and-mouth disease virion: Structure and function. Foot Mouth Dis. Virus Curr. Res. Emerg. Trends.

[46] Kotecha A., Seago J., Scott K., Burman A., Loureiro S., Ren J., Porta C., Ginn H.M., Jackson T., Pérez-Martin E. (2015). Structure-based energetics of protein interfaces guides foot-and-mouth disease virus vaccine design. Nat. Struct. Mol. Biol..

[47] Kotecha A., Wang Q., Dong X., Ilca S.L., Ondiviela M., Zihe R., Seago J., Charleston B., Fry E.E., Abrescia N.G.A. (2017). Rules of engagement between alphavbeta6 integrin and foot-and-mouth disease virus. Nat. Commun..

[48] Duque H., Baxt B. (2003). Foot-and-mouth disease virus receptors: Comparison of bovine αv integrin utilization by type A and O viruses. J. Virol..

[49] Zhang Y., Sun Y., Yang F., Guo J., He J., Wu Q., Cao W., Lv L., Zheng H., Zhang Z. (2013). Induction of partial protection against foot and mouth disease virus in guinea pigs by neutralization with the integrin beta6-1 subunit. Viruses.

[50] Miller L.C., Blakemore W., Sheppard D., Atakilit A., King A.M., Jackson T. (2001). Role of the cytoplasmic domain of the beta-subunit of integrin alpha(v)beta6 in infection by foot-and-mouth disease virus. J. Virol..

[51] Xu X., Nagarajan H., Lewis N.E., Pan S., Cai Z., Liu X., Chen W., Xie M., Wang W., Hammond S. (2011). The genomic sequence of the Chinese hamster ovary (CHO)-K1 cell line. Nat. Biotechnol..

[52] Lawrence P., Rieder E. (2017). Insights into Jumonji C-domain containing protein 6 (JMJD6): A multifactorial role in foot-and-mouth disease virus replication in cells. Virus Genes.

[53] Lee G., Hwang J.-H., Kim A., Park J.-H., Lee M.J., Kim B., Kim S.-M. (2020). Analysis of amino acid mutations of the foot-and-mouth disease virus serotype O using both heparan sulfate and JMJD6 receptors. Viruses.

[54] Zhao Q., Pacheco J.M., Mason P.W. (2003). Evaluation of genetically engineered derivatives of a Chinese strain of foot-and-mouth disease virus reveals a novel cell-binding site which functions in cell culture and in animals. J. Virol..

[55] Maree F., Blignaut B., De Beer T.A., Visser N., Rieder E.A. (2010). Mapping of amino acid residues responsible for adhesion of cell culture-adapted foot-and-mouth disease SAT type viruses. Virus Res..

[56] Dill V., Hoffmann B., Zimmer A., Beer M., Eschbaumer M. (2018). Influence of cell type and cell culture media on the propagation of foot-and-mouth disease virus with regard to vaccine quality. Virol. J..

[57] International Office of Epizooties (1990). Outerbreaks during preceding months. OIE Bull..

[58] Li P., Lu Z., Bai X., Li D., Sun P., Bao H., Fu Y., Cao Y., Chen Y., Xie B. (2014). Evaluation of a 3A-truncated foot-and-mouth disease virus in pigs for its potential as a marker vaccine. Veter. Res..

[59] Yuan H., Li P., Bao H., Sun P., Bai X., Bai Q., Li N., Ma X., Cao Y., Fu Y. (2020). Engineering viable foot-and-mouth disease viruses with increased acid stability facilitate the development of improved vaccines. Appl. Microbiol. Biotechnol..

[60] Fu Y., Li P., Cao Y., Wang N., Sun P., Shi Q., Ji X., Bao H., Li D., Chen Y. (2017). Development of a blocking ELISA using a monoclonal antibody to a dominant epitope in non-structural protein 3A of foot-and-mouth disease virus, as a matching test for a negative-marker vaccine. PLoS ONE.

